# The Neuroprotective Effect of Thalidomide against Ischemia through the Cereblon-mediated Repression of AMPK Activity

**DOI:** 10.1038/s41598-018-20911-2

**Published:** 2018-02-06

**Authors:** Naoya Sawamura, Mariko Yamada, Miku Fujiwara, Haruka Yamada, Hideki Hayashi, Norio Takagi, Toru Asahi

**Affiliations:** 1 Faculty of Science and Engineering, Waseda University, TWIns, 2–2 Wakamatsu, Shinjuku, Tokyo 162–8480 Japan; 2 Research Organization for Nano & Life Innovation, Waseda University, #03C309, TWIns, 2–2 Wakamatsu, Shinjuku, Tokyo 162–8480 Japan; 3 Department of Applied Biochemistry, Tokyo University of Pharmacy and Life Sciences, 1432–1 Horinouchi, Hachioji, Tokyo 192–0392 Japan

## Abstract

Thalidomide was originally used as a sedative and found to be a teratogen, but now thalidomide and its derivatives are widely used to treat haematologic malignancies. Accumulated evidence suggests that thalidomide suppresses nerve cell death in neurologic model mice. However, detailed molecular mechanisms are unknown. Here we examined the molecular mechanism of thalidomide’s neuroprotective effects, focusing on its target protein, cereblon (CRBN), and its binding protein, AMP-activated protein kinase (AMPK), which plays an important role in maintaining intracellular energy homeostasis in the brain. We used a cerebral ischemia rat model of middle cerebral artery occlusion/reperfusion (MCAO/R). Thalidomide treatment significantly decreased the infarct volume and neurological deficits of MCAO/R rats. AMPK was the key signalling protein in this mechanism. Furthermore, we considered that the AMPK–CRBN interaction was altered when neuroprotective action by thalidomide occurred in cells under ischemic conditions. Binding was strong between AMPK and CRBN in normal SH-SY5Y cells, but was weakened by the addition of H_2_O_2_. However, when thalidomide was administered at the same time as H_2_O_2_, the binding of AMPK and CRBN was partly restored. These results suggest that thalidomide inhibits the activity of AMPK via CRBN under oxidative stress and suppresses nerve cell death.

## Introduction

Cerebral infarction is a disease in which the brain becomes oxygen deficient and energy deficient due to stenosis and obstruction of cerebral blood vessels, causing brain cell damage and ultimately leading to nerve cell death. Cerebral infarction is one type of stroke, known as ischemic stroke. Cerebral infarction is a major cause of death worldwide and causes various severe disorders, so discovery of an effective treatment is greatly needed.

Thalidomide is a teratogen known for inducing birth defects, but now thalidomide and its derivatives lenalidomide and pomalidomide are widely used to treat haematologic malignancies including multiple myeloma (MM) and 5q deletion associated myelodysplastic syndrome (del(5q)MDS). Several groups have reported the effect of thalidomide on neuronal cells, including neuronal damage resulting from focal cerebral ischemia^1^ and amyloid beta-induced impairment of recognition memory^2^. Furthermore, it has been reported that thalidomide has a protective effect in a rat cerebral ischemic model, by reducing both oxidative stress and inflammatory response^3^. However, the detailed molecular mechanism of thalidomide’s neuroprotective effect has not yet been elucidated.

*Cereblon* (*CRBN*), on chromosome 3p26.2, is a gene responsible for mild and severe autosomal recessive non-syndromic intellectual disabilities^4^. CRBN is also identified as a target protein of thalidomide^5^. CRBN functions as a substrate receptor of the Cullin4 (Cul4)-DDB1 E3 ubiquitin ligase complex. It has been suggested that thalidomide binds to CRBN, thereby impairing CRBN’s activity as an E3 ubiquitin ligase complex and causing teratogenicity^5^. Thalidomide and its analogues associate with CRBN to support proteasome-dependent degradation of the transcription factors IKZF1 (Ikaros) and IKZF3 (Aiolos), and Casein kinase 1 alfa (CSNK1A1)^6–9^. IKZF1 and IKZF3 are both essential in MM, and CSNK1A1 is thought to be a molecular target of del(5q)MDS. This leads to growth inhibition and apoptosis of MM cells following downregulation of c-Myc and IRF4^7,9–12^. Altogether, previous reports show that CRBN functions in the alteration of ubiquitination and degradation of specific targets.

Expression of CRBN protein has been confirmed throughout the entire body, particularly in the cerebrum, and is widely present in various organelles such as cytoplasm, nucleus, mitochondria, and endoplasmic reticulum^13^. In our previous papers, we elucidated the functions of CRBN in each organelle. In cytoplasm, CRBN forms aggresomes near the nucleus in the presence of proteasome inhibitor and protects against cell death under proteasome stress^14^. In the nucleus, CRBN modulates the activity of the transcription factor Ikaros, and thus the expression level of its downstream target, encephalin^15^. Furthermore, mitochondrial CRBN has a cytoprotective effect against oxidative stress^16^. As described above, it has been suggested that CRBN is a multi-functional, stress response protein.

AMP-activated protein kinase (AMPK) has been identified as a CRBN binding protein^17^. AMPK is an important intracellular energy sensor, a protein that is deeply involved in energy metabolism in the brain^18^. AMPK is a heterotrimer composed of α, β and γ subunits. AMPK is activated by phosphorylation of threonine at position 172 (Thr 172) of the α subunit^19,20^. Activation of AMPK occurs when cells are exposed to various stresses such as ATP depletion, oxidative stress, low glucose, or ischemia^21–23^. This induces ATP production and maintains homeostasis of intracellular ATP levels^24^. Because the AMPK α subunit binds to CRBN, it has been reported that CRBN dephosphorylates AMPK and suppresses its activity^17^. Previous studies have also shown that AMPK undergoes phosphorylation and amplification as a result of cerebral ischemia, and suppression (dephosphorylation) of AMPK activity is thought to lead to neuroprotection^25,26^.

Here we examined the molecular mechanism of thalidomide’s neuroprotective effect on cerebral infarction, focusing on its target protein, CRBN, and its binding protein, AMPK. Our results suggest that thalidomide inhibits the activity of AMPK via CRBN under oxidative stress and suppresses nerve cell death caused by cerebral ischemia.

## Results

### The effect of thalidomide on infarct lesions caused by cerebral ischemia

**Figure 1:**
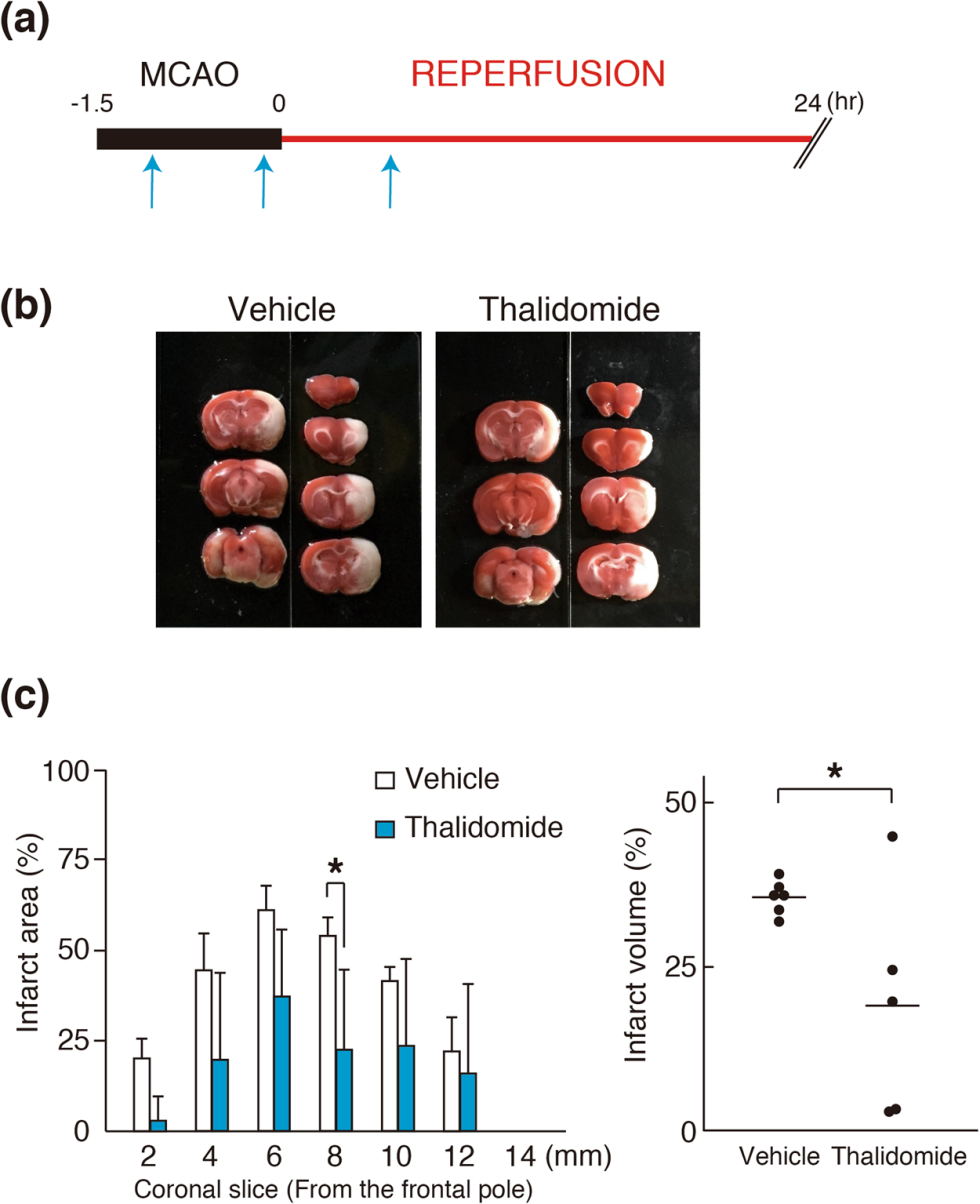
The effect of thalidomide on infarct volume of the MCAO/R model. (**a**) Experimental design. Animals underwent 1.5 h of middle cerebral artery occlusion and 24 h reperfusion time (MCAO/R). Thalidomide (20 mg/kg) or DMSO (0.66 ml/kg, vehicle) administration is shown in asterisks. Sample preparation for histological, neurological, and biochemical studies were performed at the end of reperfusion. (**b**) Representative photographs of TTC-stained brain slices showing the infarct area 24 h after MCAO/R. (**c**) Quantitative analysis of the infarct area in each slice and the total infarct volume from MCAO/R rats treated with thalidomide or vehicle. The infarct areas caused by MCAO/R were significantly decreased by the administration of thalidomide. Quantification of the infarct volume is represented by mean ± SD (n = 6 for vehicle, n = 5 for thalidomide).

The effect of thalidomide on infarct lesions caused by cerebral ischemia was examined using cerebral ischemia model rats (Fig. 1a). 2,3,5-Triphenyltetrazolium chloride (TTC) staining was performed to measure infarct lesions after middle cerebral artery occlusion/reperfusion (MCAO/R) (Fig. 1b). Quantitative analysis showed that thalidomide significantly reduced the infarct areas in slice 4 compared with the vehicle group (Fig. 1c). Calculating the total volume of each slice revealed that the infracted area caused by MCAO/R was significantly decreased by thalidomide treatment (*p < 0.05, Fig. 1c). These results suggest that thalidomide administration reduced the infarct area, supporting that thalidomide is effective for cerebral ischemia^1,3,27^.

### Effect of thalidomide on neurological deficits caused by cerebral ischemia

**Figure 2:**
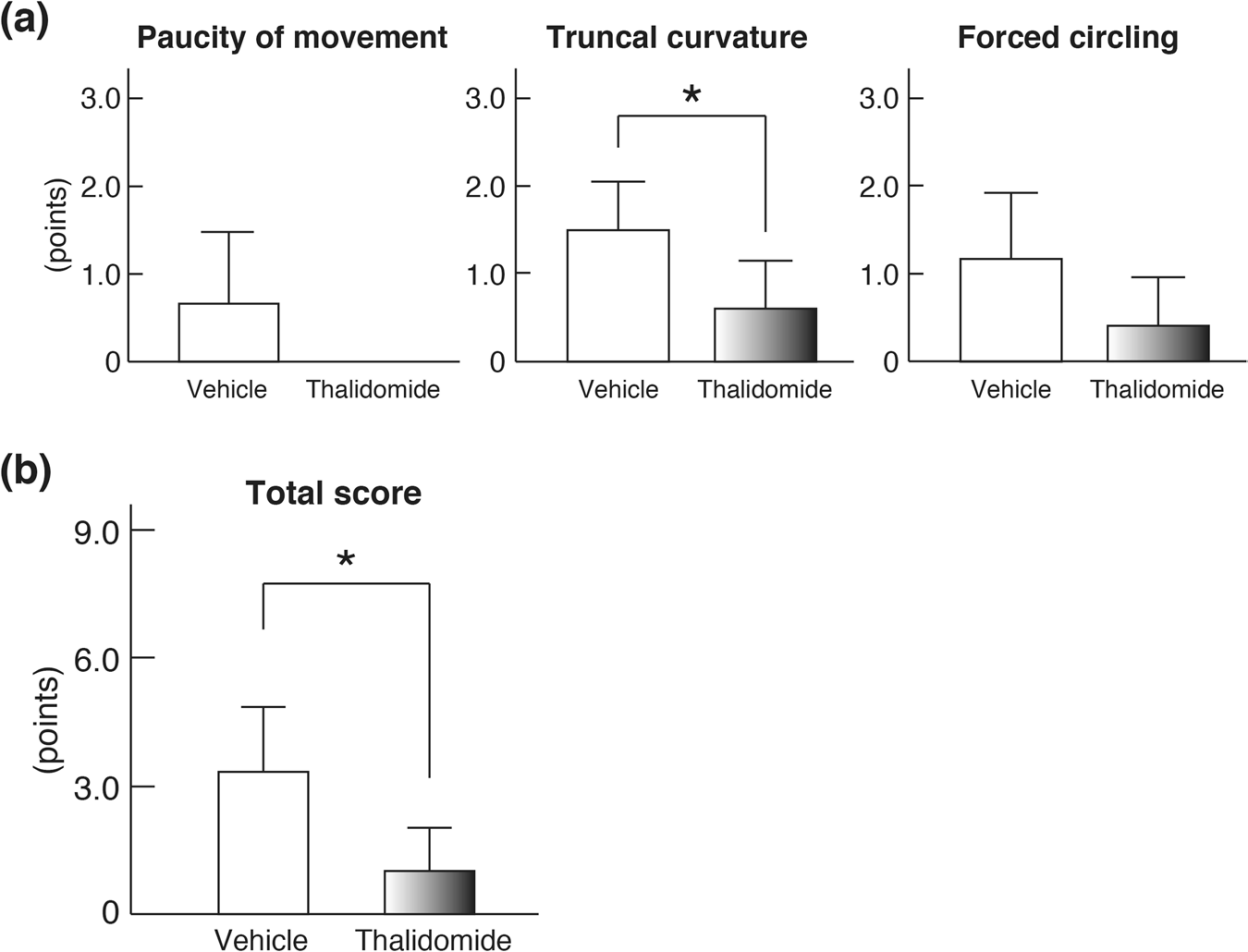
The effect of thalidomide on neurological deficits of the MCAO/R model. (**a**) Assessments of neurological deficits were performed at 24 h after MCAO/R using the modified Bederson’s method^36^. In the right hemisphere MCAO/R model, motor dysfunction occurred in the left side of the body that was the dominant region of the right cerebral hemisphere. Scoring was performed on three items: paucity of movement, forced circling during locomotion, and truncal curvature. The scoring system was: 3 points for high severity, 2 points for medium level, 1 point for small degree, and 0 points for asymptomatic; the points for each item were compared. The score of truncal curvature resulting from the cerebral infarction by MCAO/R was significantly reduced by the administration of thalidomide (*p < 0.05). (**b**) Total points of the three items were compared. Neurological deficits resulting from the cerebral infarction by MCAO/R were significantly reduced by the administration of thalidomide (*p < 0.05).

We next examined how thalidomide administration affects the neurological deficit symptoms caused by cerebral ischemia. When assessed for paucity of movement, forced circling during locomotion, and truncal curvature, the average score of symptom severity in cerebral ischemic rats treated with thalidomide was lower than that of vehicle-treated rats for all three measures. (Fig. 2a). Regarding the total score (full score of 9 points), cerebral ischemic rats treated with thalidomide recorded significantly lower values than vehicle-treated rats (*p < 0.05, Fig. 2b). These results further support that thalidomide is effective for cerebral ischemia, as thalidomide administration reduced the neurological deficiency symptoms of cerebral ischemic rats.

### AMPK is the key signalling protein participating in thalidomide-induced neuroprotection in ischemia

Several different signalling cascades (e.g., ERK1/2, AMPK, and the AKT pathway) have been reported to participate in neuronal cell survival^18,28–30^. Therefore, we investigated the activation of these signalling proteins to identify which proteins participate in thalidomide-induced neuroprotection in ischemia.

**Figure 3:**
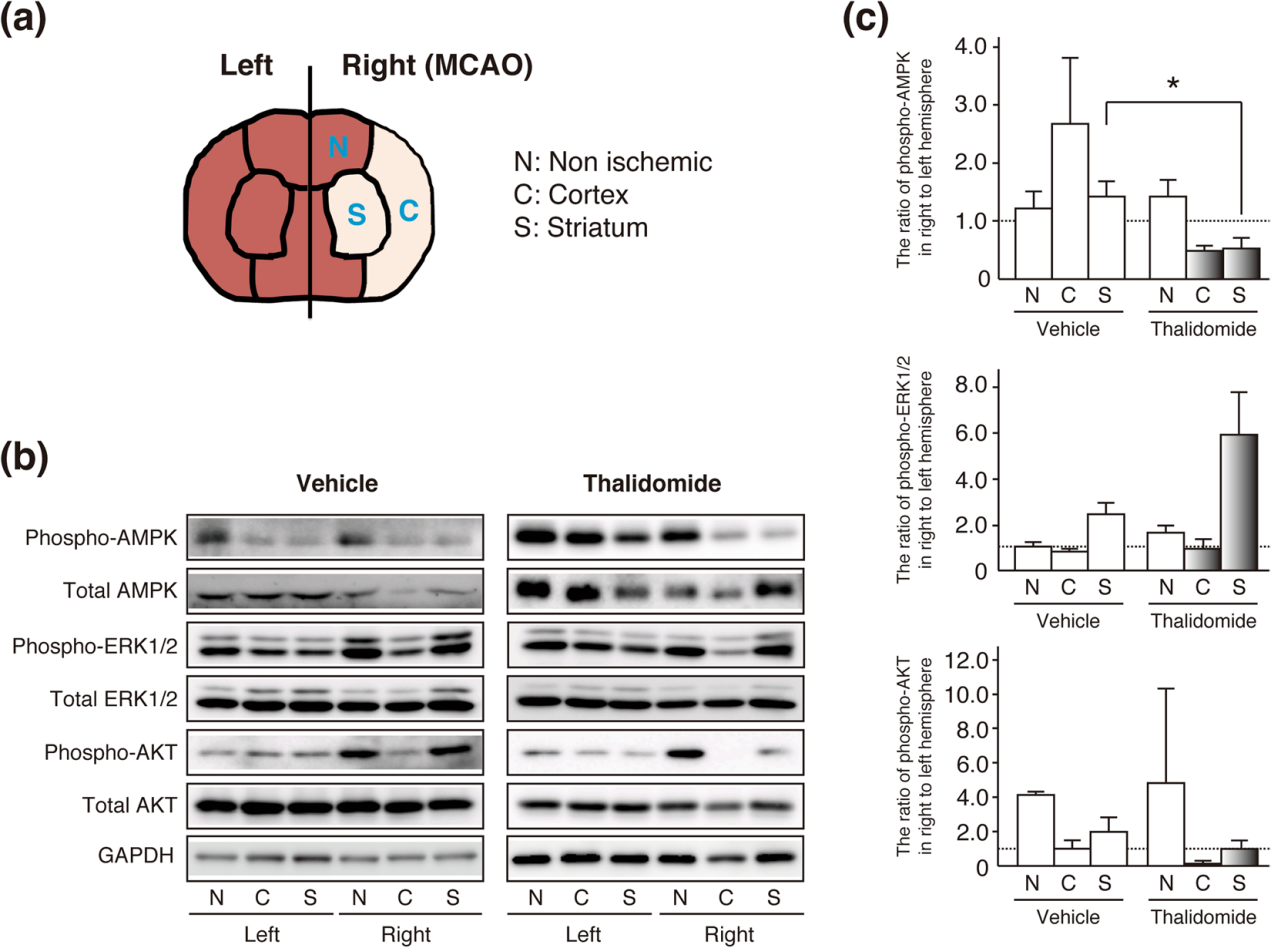
The effect of thalidomide on neuroprotective signalling molecules in the MCAO/R model. (**a**) Schematic drawing of brain regions used in the experiments. (**b**,**c**) Western blotting was carried out for each region, and phosphorylation (activation) of AMPK, ERK1/2, and AKT was detected and quantified. The data were normalized as the ratio of the right hemisphere (ischemic) to left hemisphere in thalidomide-treated or vehicle rats. No difference in signalling protein activation was observed in non-ischemic (N) and ischemic cortex (C) regions between the vehicle and thalidomide-treated groups. However, amplification of phosphorylation due to cerebral ischemia was significantly suppressed in the striatum (S) by thalidomide administration (*p < 0.05). Cropped blots are displayed and full-length blots are presented in Supplementary Fig. 1. Quantification of western blots for all signalling proteins is represented by mean ± SD (n = 3).

Western blotting was carried out for each region (Fig. 3a), and phosphorylation (activation) of AMPK, ERK1/2, and AKT was detected and quantified. AMPK phosphorylation was amplified by cerebral ischemia, but was not amplified in the thalidomide-treated group. In particular, amplification of phosphorylation due to cerebral ischemia was significantly suppressed in the striatum by thalidomide administration (*p < 0.05, Fig. 3b,c). Therefore, the results show that administration of thalidomide suppresses AMPK phosphorylation amplification during cerebral ischemia. Thalidomide had no effect on ERK1/2 or AKT (Fig. 3b,c).

These results suggest that AMPK may play a role as an intracellular signal in the thalidomide-induced nerve cell death suppression effect.

### The effect of thalidomide on neuronal death in cerebral ischemia model cells

**Figure 4:**
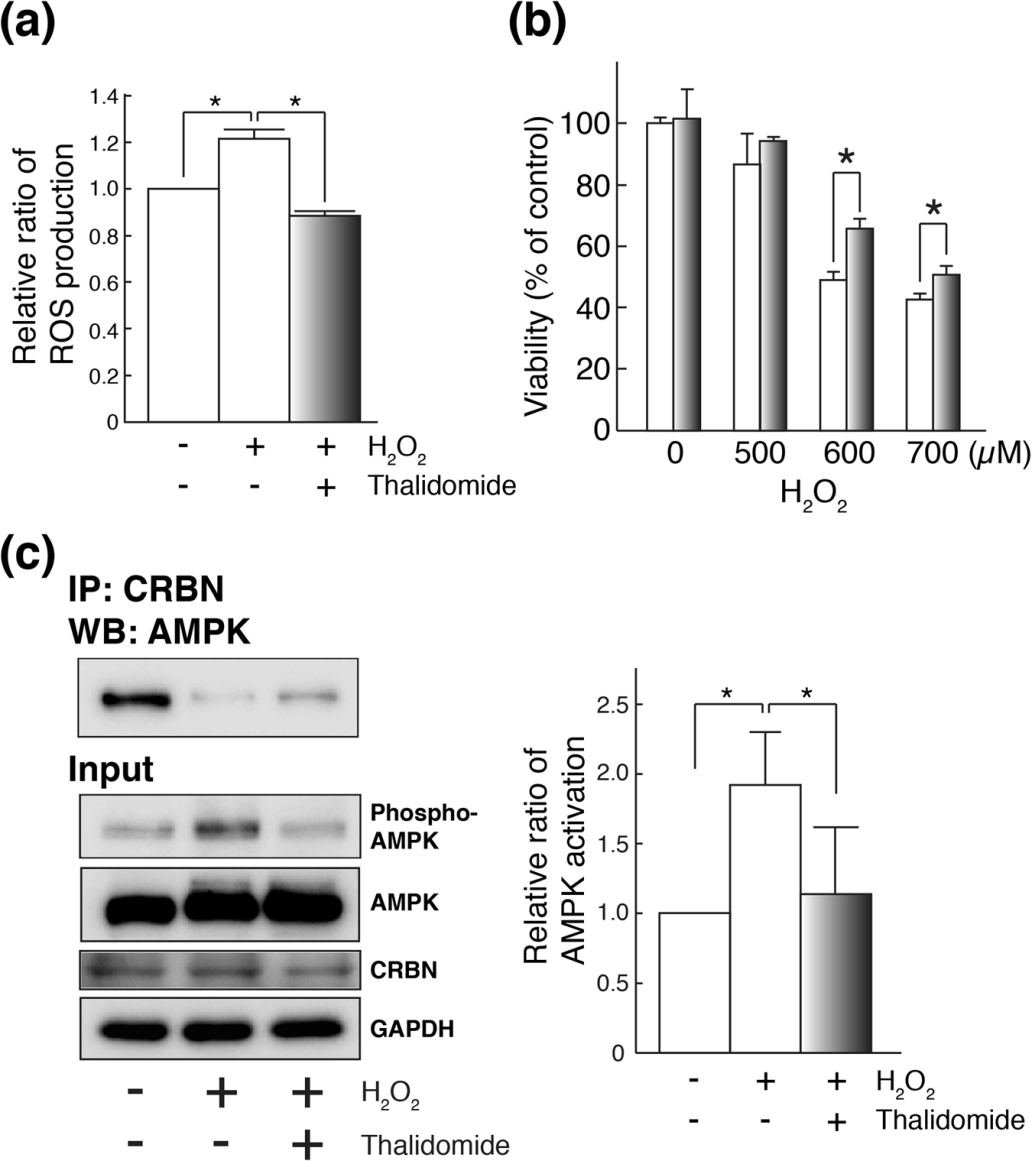
Molecular mechanism of thalidomide on AMPK activation in H_2_O_2_-treated SH-SY5Y cells. (**a**) Generation of intracellular reactive oxygen species (ROS) in SH-SY5Y cells was measured using the ROS-sensitive fluorescent dye, CM-H_2_DCFDA. Thalidomide treatment significantly suppressed ROS production induced by H_2_O_2_ in SH-SY5Y cells (*p < 0.05). Quantification of ROS production is represented by mean ± SD (n = 3). (**b**) H_2_O_2_ (500, 600, 700 µM) was added to SH-SY5Y cells to induce neuronal cell death. Thalidomide (10 µM) treatment significantly (*p < 0.05) suppressed neuronal cell death induced by oxidative stress. Quantification of the MTS assay is represented by mean ± SD (n = 3). (**c**) AMPK activation in H_2_O_2_-treated SH-SY5Y cells and the repression of the AMPK activation were confirmed (*p < 0.05, input sample). Co-immunoprecipitation analysis showed that CRBN interacts with AMPK under physiological conditions. The binding between AMPK and CRBN was weakened when H_2_O_2_ was added. However, when thalidomide was added at the same time as H_2_O_2_, the binding between AMPK and CRBN was partly restored. Cropped blots are displayed and full-length blots are presented in Supplementary Fig. 2.

To determine the molecular mechanism, the effect of thalidomide was examined on ischemia model cells. Oxidative stress-induced neuronal cells were used as ischemia model cells. Oxidative stress was induced in human neuroblastoma SH-SY5Y cells by administration of H_2_O_2_, with or without thalidomide treatment, and intracellular reactive oxygen species (ROS) were measured to examine the effect of thalidomide on ROS generation. When SH-SY5Y cells were treated with H_2_O_2_, the amount of ROS increased. However, when thalidomide was administered at the same time, the fluorescence intensity significantly decreased (Fig. 4a). In order to relate with the H_2_O_2_-induced damage in the cell culture, we observed the level of the advanced glycation end products (AGEs), a marker of oxidative stress, in MCAO/R. The levels of AGEs tended to be elevated in the striatum of vehicle rat, and it was suppressed in the striatum of thalidomide-treated rat (Supplementary Fig. 3). Next, the effect of thalidomide on cell viability was measured. When H_2_O_2_ was added to SH-SY5Y cells, the cell viability decreased depending on the H_2_O_2_ concentration. However, when thalidomide was added at the same time as H_2_O_2_, the survival rate was significantly higher than for those treated with H_2_O_2_ only, and the neuronal cell death induced by H_2_O_2_ was significantly suppressed (*p < 0.05, Fig. 4b). These results suggest that thalidomide is effective for cerebral ischemia model cells in addition to cerebral ischemic model rats.

### Molecular mechanism of the neuroprotective effect by thalidomide—Inhibition of AMPK activity via thalidomide target protein CRBN

The molecular mechanism of thalidomide-induced nerve cell protection was examined in detail by focusing on the activity of AMPK modulated by its binding protein CRBN using the cerebral ischemia model cells described above. First, the activity of AMPK in cerebral ischemia model cells was examined. H_2_O_2_ treatment increased phosphorylation of AMPK in the SH-SY5Y cells. However, when thalidomide was added, phosphorylation of AMPK was suppressed, similar to the effect in the thalidomide-treated ischemia model rats (*p < 0.05, Fig. 4c). Next, immunoprecipitation was used to verify the interaction between AMPK and CRBN in the SH-SY5Y cells. AMPK and CRBN were strongly bound in normal SH-SY5Y cells. When H_2_O_2_ was added, the binding between AMPK and CRBN weakened. However, when thalidomide was added at the same time as H_2_O_2_, the binding between AMPK and CRBN was partly restored (Fig. 4c).

These results suggest that in cerebral ischemia model cells, the AMPK–CRBN interaction is weakened and phosphorylation of AMPK is enhanced, but thalidomide treatment restores the AMPK–CRBN interaction and suppresses phosphorylation of AMPK.

## Discussion

In this study, we investigated the effect of thalidomide on the neurological deficiency symptoms caused by cerebral ischemia. Cerebral ischemic rats treated with thalidomide had lower scores than vehicle-treated rats in paucity of movement, forced circling during locomotion, and truncal curvature. Cerebral ischemic rats treated with thalidomide also recorded significantly lower total scores than controls, indicating that the nerve deficit symptoms were significantly reduced. In addition, cerebral ischemic rats demonstrated symptoms other than the three measured nerve deficit symptoms, including one-way lifting of the head during tail suspension. This is because, similar to the effect on turning, movement was impaired only on the opposite side of the injection. Furthermore, in the thalidomide-treated cerebral ischemic rats, many sleeping rats were observed about 1 h after administration. This is thought to be due to the hypnotic action of thalidomide. Therefore, to investigate the effect of thalidomide on nerve deficiency symptoms in this experiment, scoring was performed about 24 h after administration.

To investigate intracellular signals involved in thalidomide suppression of neuronal cell death, we detected AMPK, ERK1/2, and Akt, which are neuroprotective signalling molecules. No significant activation change was observed with ERK1/2 or Akt by thalidomide administration. The activation of both PI3K/AKT pathway and ERK1/2 pathways has been previously related with neuroprotection effect^31,32^. Both of them are known as insulin-dependent signaling pathways. Recent studies suggest that CRBN regulates organismic growth via insulin dependent signaling pathways. *CRBN*-deficient mice showed the activation of AMPK and resistance to high-fat diet-induced obesity and insulin resistance^33^. We also identified that Ohgata (OHGT), the *Drosophila* ortholog of CRBN as a novel regulator of insulin-dependent organismic growth in *Drosophila*^34^. It has a possibility that CRBN directly regulates insulin-dependent signaling pathway in the molecular mechanism of thalidomide’s neuroprotective effect on cerebral infarction. It was also reported that the involvement of PI3K/Akt signaling pathway in the mechanism of neuroprotection of thalidomide on hypoxic–ischemic cortical neurons *in vitro*^29^. The interaction of AMPK and PI3K/Akt signaling pathway in the mechanism need to be investigated.

AMPK was activated by cerebral ischemia, however, when thalidomide was administered, phosphorylation of AMPK was reduced. This is consistent with reports that suppression of AMPK activity leads to neuroprotection^25,26^. Because AMPK binds to CRBN, which is a target molecule of thalidomide^17^, thalidomide exerts a greater effect on AMPK than other signalling molecules, resulting in the most remarkable change. The strong AMPK–CRBN interaction was confirmed in the physiological condition, and AMPK was suppressed. Consistent with this result, the activation of AMPK has been reported in Crbn-deficient mice^33^. The AMPK–CRBN interaction was weakened and AMPK activation was inhibited in the cerebral ischemia models in this study. In the oxidative stress condition, expression of cytosolic CRBN, which normally binds to AMPK and suppresses its activity, is induced in mitochondria^16^, which may cause dissociation of AMPK from CRBN and activation of AMPK. However, when thalidomide is administered to cerebral ischemia models, thalidomide binds to CRBN and the AMPK–CRBN interaction might be stabilized. As a result, the AMPK–CRBN interaction partly remained, and AMPK phosphorylation was suppressed.

In addition, although AMPK phosphorylation amplification induced by cerebral ischemia was significantly suppressed in the striatum by thalidomide administration, there was a tendency for reduced AMPK activation in the cerebral cortex infarct region, but no significant difference was observed. This is because the infarct lesion caused by MCAO/R is formed so that it spreads around the striatum as the centre, thus the striatum is more seriously damaged by ischemia. The fourth slice (8–10 mm) from the rostral side, which was analysed in this study, intersects part of the striatum, so this result is considered to be valid. Because the infarct lesion is formed around the striatum, there is large variation in the extent of nerve cell death in the cerebral cortex infarct region, which is also influenced by the difference in infarct volume that is produced, so it is reasonable that no significant difference was observed.

Through this study, it was found that AMPK was activated by amplification of phosphorylation by cerebral ischemia and suppressed by thalidomide administration. It was also found that thalidomide regulates the activity of AMPK by changing the interaction of AMPK–CRBN via its target molecule CRBN. This is suggested as one of the molecular mechanisms by which thalidomide suppresses nerve cell death. However, thalidomide is an unstable drug because it undergoes hydrolysis spontaneously and rapidly in aqueous solution. Therefore, it is necessary to identify effective thalidomide derivatives with few side effects in future studies.

## Methods

### Animals

Male Sprague-Dawley rats (7 weeks old, weighing between 200 and 220 g, SLC, Shizuoka, Japan) were used in the present study. The rats were maintained at 23 ± 1 °C in a room with a constant humidity of 55 ± 5% and a 12:12 h light-dark cycle, with free access to food and water according to the Guideline for Experimental Animal Care issued by the Prime Minister’s Office of Japan. All experimental procedures were approved by the Committee of Animal Care and Welfare of Tokyo University of Pharmacy and Life Sciences.

### Animal Surgical Procedures

Transient focal ischemia was induced by the method described previously^35^ with minor modifications. Briefly, anaesthesia was induced with 5% isoflurane and maintained with 2.5% isoflurane. The surgical area was exposed, and then a 4–0 nylon surgical suture with a silicon-coated tip was inserted from the external carotid artery to the origin of the right middle cerebral artery for occlusion. After 90 min of the occlusion, the suture was removed to allow reperfusion. The behaviour of the rats was evaluated according to the method of Bederson *et al*. (1986)^36^. The rats demonstrating consistent circling toward the contralateral side and a reduced resistance to lateral push toward the contralateral side were used in the present study. Thalidomide (Sigma-Aldrich, Inc. MO, USA) was dissolved in dimethyl sulfoxide and injected intravenously, as previously reported^1^.

### Sample preparation

Seven coronal sections of the brain at 2 mm thickness were made, and the third section from the rostral side was collected for the following dissection. The coronal section was dissected into two regions, cerebral cortex and striatum (S), for both hemispheres. The cerebral cortex was further divided into the non-ischemic (N) and ischemic (C) regions. Samples of the six regions in the coronal section were defined as: LN, region of the left cerebral cortex corresponding with the non-ischemic region in the right hemisphere; LC, region of the left cerebral cortex corresponding with the ischemic region in the right hemisphere; LS, striatum in left hemisphere; RN, non-ischemic region of the cerebral cortex in right hemisphere; RC, ischemic region of the cerebral cortex in right hemisphere; and RS, striatum in right hemisphere. Dissected sections were homogenized in lysis buffer (1% Triton X-100; MP Biomedicals (Santa Ana, CA, USA), 0.1% sodium deoxycholate; Wako (Osaka, Japan), 1% EDTA; DOJINDO (Kumamoto, Japan), Complete protease inhibitor cocktail; Roche (Basel, Switzerland), PhosStop phosphatase inhibitor cocktail; Roche, in 50 mM Tris-buffered saline) with Ultrasonic Liquid Processor Q 125 (QSONICA) for 20 s at 4 °C. The homogenate was centrifuged at 15,000 × g for 5 min at 4 °C, and the supernatant was collected for immunoblotting.

### 2,3,5-Triphenyltetrazolium chloride staining and infarct size measurement

2,3,5-Tripheny-ltetrazolium chloride (TTC) staining was performed to determine the effect of thalidomide on the infarct area caused by transient focal cerebral ischemia according to the method of the previous study^35^. In brief, coronal sections of the brain at 2 mm thickness were obtained from rats treated with vehicle or thalidomide. Slices were immersed in 2% TTC/saline solution for 10 min at room temperature. Normal tissue appears red by TTC, whereas the infarct area is unstained, appearing white. The infarct area was analysed using Image J (NIH, Rockville, MD, USA).

### Antibodies

The following antibodies were used: p44/42 MAPK (ERK1/2) rabbit monoclonal antibody (Cat# 9102, RRID:AB_330744; Cell Signaling Technology, Beverly, MA, USA), phospho-p44/42 MAPK (ERK1/2; Thr202/Tyr204) rabbit monoclonal antibody (Cat# 9101, RRID:AB_331646; Cell Signaling Technology), Akt (pan) (C67E7) rabbit monoclonal antibody (Cat# 4691, RRID:AB_915783; Cell Signaling Technology), phospho-Akt (Ser473) rabbit monoclonal antibody (Cat# 9271, RRID:AB_329825; Cell Signaling Technology), and anti-phospho-AMPKα (Thr172) rabbit monoclonal antibody (Cell Signaling Technology), anti-AMPKα rabbit monoclonal antibody (Cell Signaling Technology), anti-CRBN mouse polyclonal antibody (Abnova, Taipei, Taiwan), anti-AGEs mouse monoclonal antibody (Transgenic, Inc., Kobe, Japan), anti-GAPDH mouse monoclonal antibody (Wako).

### Cell culture

Human neuroblastoma SH-SY5Y cells (ECACC Cat# 94030304, RRID:CVCL_0019) were grown in low-glucose Dulbecco’s modified Eagle’s medium (DMEM; Wako) containing 10% (v/v) fetal bovine serum and 1% penicillin-streptomycin at 37 °C with 5% CO_2_. For oxidative stress conditions, H_2_O_2_ was added to induce cytotoxicity.

### MTS cell proliferation assay

MTS cell proliferation assay was performed as described previously^37^.

### Biochemical assays

Total protein extracts were prepared from cultured cells, as described previously^38^. A standard western blotting protocol was used, as described previously^39^. Immunoprecipitation was performed as described previously^39^.

### Oxidative stress

Production of ROS after hydrogen peroxide treatment was measured. SH-SY5Y cells were seeded in 12-well plates, and pre-incubated with or without 5 µM thalidomide for 24 h. After thalidomide was washed off, cells were treated with 750 µM hydrogen peroxide for 2 h. Levels of intracellular ROS production were determined using CM-H_2_DCFDA (Life Technologies, Carlsbad, CA, USA), according to the manufacturer**’**s instructions. Fluorescence of CM-H_2_DCFDA was measured using a plate reader (Powerscan HT; DS Pharma Biomedical, Osaka, Japan).

### Statistical analysis

Measurement and quantification of data were performed by investigators blinded to treatment. Data were analysed using Student’s *t*-test, and analysis of variance followed by Dunnett’s test, with differences between samples considered statistically significant at *p* < 0.05. Data were expressed as the mean ± SD of three to six independent experiments.

### Data Availability

The datasets generated during and/or analysed during the current study are available from the corresponding author on reasonable request.

## Electronic supplementary material

Supplementary Figures
