# Supplementary material for: The Neuroprotective Effect of Thalidomide against Ischemia through the Cereblon-mediated Repression of AMPK Activity
Source: Sci Rep. 2018 Feb 6;8:2459. doi: 10.1038/s41598-018-20911-2 (PMC5802741; doi:10.1038/s41598-018-20911-2)
Supplement: Supplementary file 1 — Supplementary Figures [file 41598_2018_20911_MOESM1_ESM.pdf]

Supplementary information

The Neuroprotective Effect of Thalidomide against Ischemia through the Cereblon-mediated Repression of AMPK Activity

Naoya Sawamura[a,b,*], Mariko Yamada[c], Miku Fujiwara[a], Haruka Yamada[a],

Hideki Hayashi[c], Norio Takagi[c], and Toru Asahi[a,b]

[a]Faculty of Science and Engineering, Waseda University, TWIns, 2-2 Wakamatsu, Shinjuku, Tokyo 162-8480, Japan

[b]Research Organization for Nano & Life Innovation, Waseda University,

#03C309, TWIns, 2-2 Wakamatsu, Shinjuku, Tokyo 162-8480, Japan

[c]Department of Applied Biochemistry, Tokyo University of Pharmacy and Life Sciences, 1432-1 Horinouchi, Hachioji, Tokyo 192-0392, Japan

*Corresponding Author:
Naoya Sawamura, Ph.D.
Associate Professor
Research Organization for Nano & Life Innovation, Waseda University
#03C309, TWIns, 2-2 Wakamatsu, Shinjuku, Tokyo 162-8480, Japan.
Tel./Fax: +81-3-5369-7327
E-mail: naoya.sawamura@gmail.com

Supplementary Information

Supplementary Figure 1, 2. Full-length immunoblots related to Fig. 3, 4.

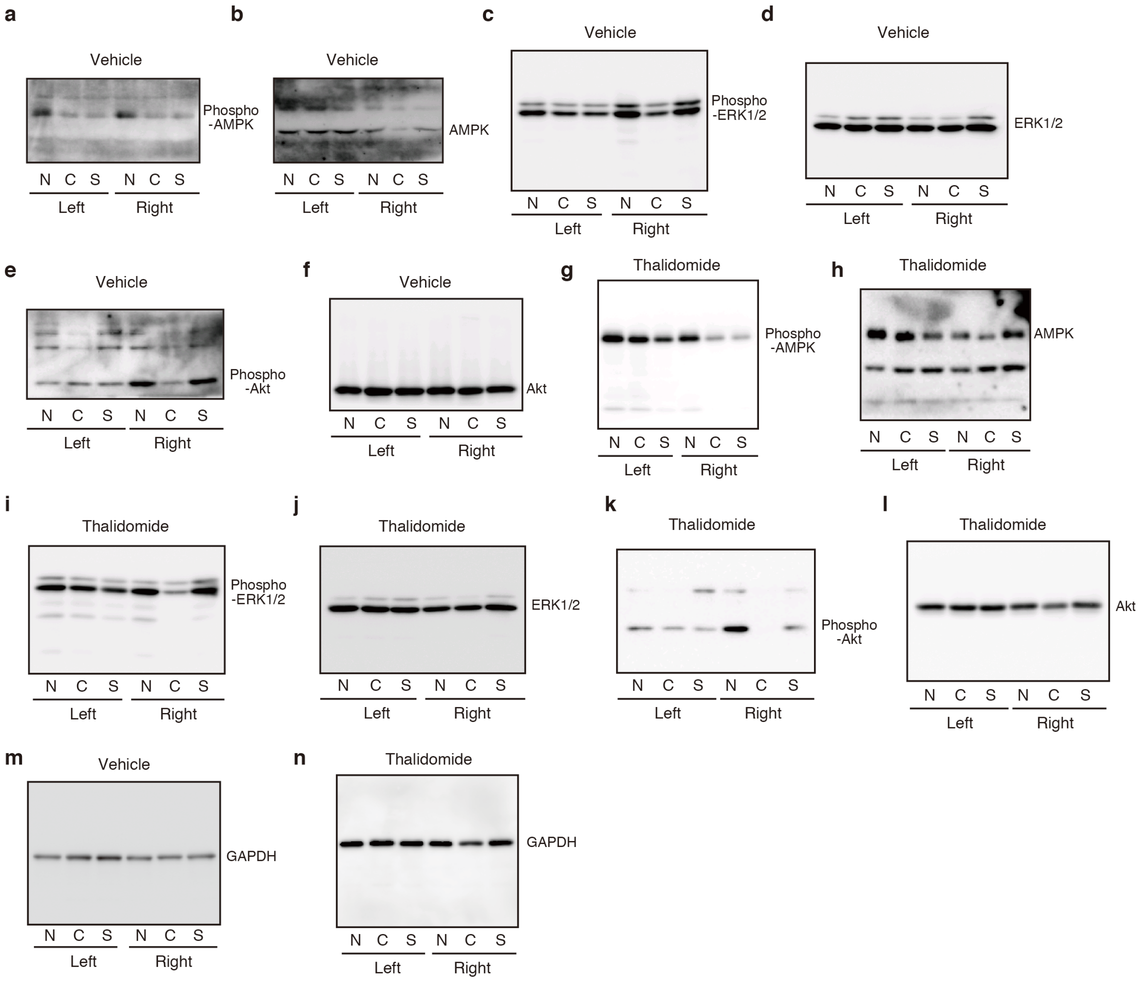

Supplementary Figure 1. Full-length immunoblots related to Fig. 3. (a-n) Full-length immunoblots relating to Fig. 3 are shown.

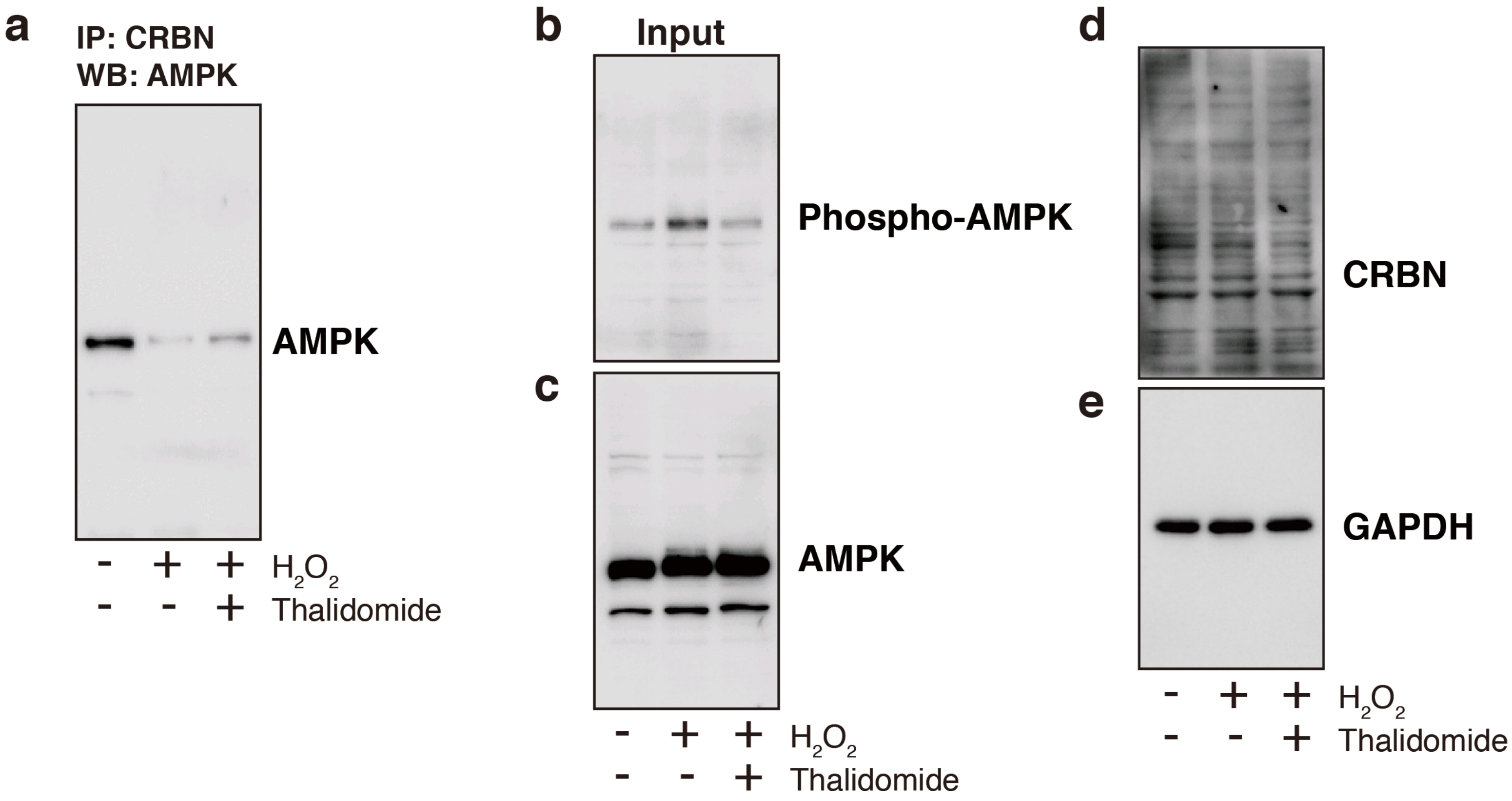

Supplementary Figure 2. Full-length immunoblots related to Fig. 4. (a-e) Full-length immunoblots relating to Fig. 4 are shown.

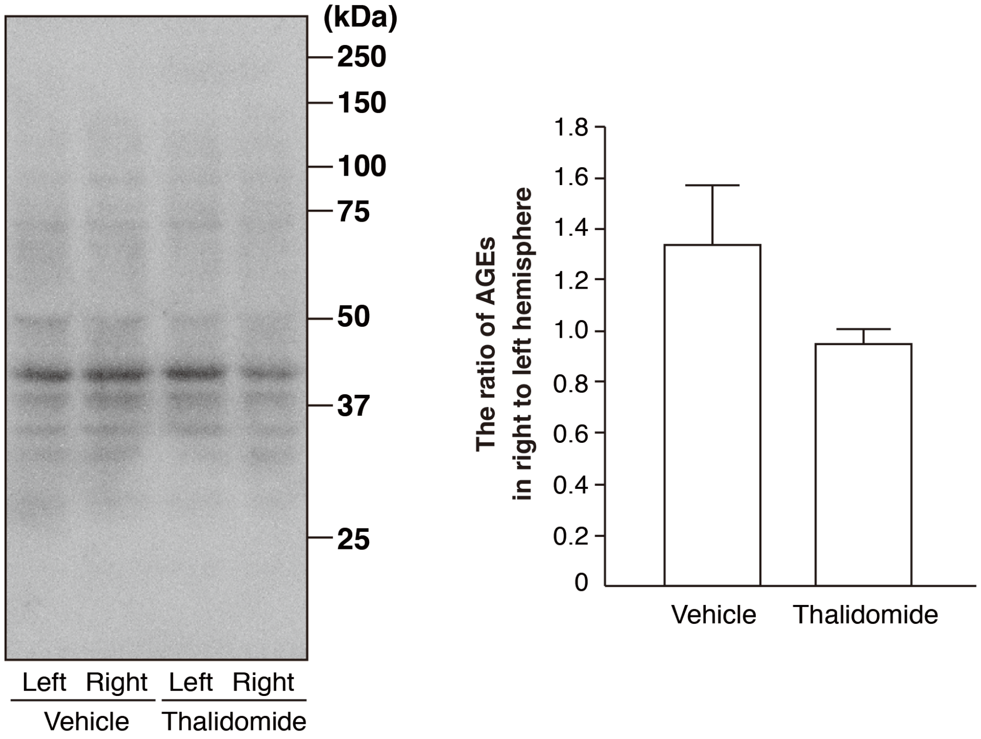

Supplementary Figure 3. The level of the advanced glycation end products (AGEs), a marker of oxidative stress, was detected by western blotting using antibody against AGEs. AGEs produced by cerebral ischemia were increased in vehicle group. The levels of AGEs due to cerebral ischemia tend to be suppressed by thalidomide administration.
